# Use of VetCompass Australia data to estimate body weight for antimicrobial stewardship in Australian dogs

**DOI:** 10.1111/avj.70099

**Published:** 2026-06-04

**Authors:** L Hardefeldt, K Li, K Bailey

**Affiliations:** ^1^ Asia‐Pacific Centre for Animal Health, Melbourne Veterinary School & National Centre for Antimicrobial Stewardship University of Melbourne Parkville Victoria Australia

**Keywords:** antimicrobial resistance, big data, canine

## Abstract

Evaluation of the appropriateness of antimicrobial use requires multiple data elements, including the indication for treatment, duration of therapy and dose of the drug used. To calculate the dose the volume of active ingredient and animal weight are required. Dog weight is frequently missing from the electronic medical records from the VetCompass Australia database. Electronic clinical records were extracted from VetCompass Australia for the top 10 breeds of dogs in the database where weight, age and sex were recorded (n = 72,089). The mean weight for age and sex was calculated for each breed. In a second cohort, animals aged greater than 1 year that had weight recorded and were prescribed an antimicrobial were extracted (n = 69,683). The total milligrams of antimicrobial administered were extracted using previously described tools. The true weight and the breed/sex/age average were then used to assess the appropriateness of antimicrobial doses. Antimicrobial doses were compared to those recommended in the Australian Veterinary Prescribing Guidelines from the University of Melbourne. There were 46,172 appropriate doses of antimicrobials in cohort 2 (66% appropriateness). The true positive rate for VetCompass Australia approximated weight was 82%. The true negative rate for VetCompass Australia approximated weight was 78%. Subgroup mean imputation is a reasonably accurate method of dealing with missing data for determining the appropriateness of antimicrobial dose for population level studies.

**Abbreviation**
AKC: American Kennel Club

Antimicrobials are widely used across both human and veterinary medicine to treat and prevent infections. Inappropriate use, such as under‐ and overdosing, can lead to serious consequences such as the development of resistance^1^ and impact patient safety and treatment efficacy.^2^, ^3^ Antimicrobial stewardship programs are increasingly implemented in veterinary practices to improve the appropriate use of antimicrobials.^4^, ^5^, ^6^ Evaluation of the appropriateness of antimicrobial use requires assessment of several elements of a prescription including the indication for use, the agent administered, the duration of use and the dosing regimen utilised. Evaluation of the appropriateness of use is widespread in human hospitals around the world using a point prevalence survey – the National Antimicrobial Prescribing Survey^7^, ^8^ however, data collection is manual and time‐consuming. Evaluation of electronic veterinary records using natural language processing tools represents an opportunity to reduce the cost of performing audits and program evaluation. However, all the required elements for evaluating appropriateness of use were found in less than 40% of electronic veterinary records.^9^ Patient weight was missing in 59% of records, meaning the dosing regimen could not be extrapolated.

Missing data is a common problem in research involving electronic medical records. Multiple imputation aims to reflect uncertainty about missing values by creating multiple plausible completed datasets and combining the results. Subgroup mean imputation is a simple, single‐imputation approach that replaces missing values with the observed mean within a defined subgroup. It may underestimate variability, but it may be useful for prospective audit and feedback due to its simplicity and ease of use.

This study aimed to determine the average weight of common dog breeds in Australia, and then evaluate the accuracy of using subgroup mean imputation for weight, and imputing using weights published by the American Kennel Club (AKC),^10^ for assessing dose appropriateness for canine drug prescriptions. The AKC was used as a source of breed standards for purebred dogs as it includes approximate weight ranges, whereas not all Australian breed standards do. Our hypothesis was that imputed body weights from VetCompass Australia would have acceptable accuracy compared to actual weight for use in assessing appropriateness of antimicrobial drug dosing in common dog breeds in Australia.

## Materials and methods

### Cohort 1

Electronic clinical records were extracted from VetCompass Australia^11^ for the top 10 breeds of dogs in the database where weight, age and sex were recorded. The top 10 breeds were identified by searching the ‘Patient Breeds’ and allowing for alternative common breed names (i.e. Australian Cattle Dog, Blue Heeler, Red Heeler all map to Australian Cattle Dog). Breeds with different sizes, such as Poodles (i.e. miniature, toy, standard etc), were excluded as were crossbreeds such as Groodles. Records spanned from 21 November 1999 to 4 August 2023. Only the first record for each patient was included. The official breed weight was obtained from weight information published by the AKC (Table 1). Records with body weight greater than 3 standard deviations from the mean were excluded. AKC weights were compared to VetCompass mean weights using a one sample t‐test.

**Table 1:** American Kennel Club indicative breed weights for adult dogs

AKC breed name	Sex	Range (kg)	Mean (kg)
Australian cattle DAustralian cattle Dogog	A	16–23	19.5
Australian kelpie	A	16–23	19.5
Border collie	A	14–25	19.5
Cavalier king Charles spaniel	A	6–8	7
German shepherd dog	F	23–32	27.5
	M	29–41	35
Golden retriever	F	25–29	27
	M	29–34	31.5
Labrador retriever	F	25–32	28.5
	M	29–36	32.5
Rottweiler	F	36–45	40.5
	M	43–61	52
Staffordshire bull terrier	F	11–15	13
	M	13–17	15

Jack Russel Terriers are not present in American Kennel Club database. A, all; F, female; M, male.

Age was treated as a categorical variable and rounded to the nearest whole number of years. Multiple imputation of weight was performed using multivariate normal regression and the Stata mi suite. The impact of factors including sex, age and neutered status on weight, stratified by breed, was investigated using a multivariable generalised linear regression model. All statistical analysis was performed in Stata (StatCorp, Tx, version 17).

### Cohort 2

Electronic clinical records were extracted from VetCompass Australia for dogs in the top 10 breeds, aged greater than 1 year, that had weight recorded and were prescribed an antimicrobial. Records spanned from 12 January 2000 to 10 July 2023. Records with body weight greater than 3 standard deviations from the mean were excluded. Records that included an antimicrobial without a dose recommendation in the Australian Veterinary Prescribing Guidelines from the University of Melbourne^12^ were excluded. Antimicrobials that were administered via an injection (IV, IM and SC) were also excluded. The total milligrams of antimicrobial administered were extracted using previously described tools.^9^ The dosing regimen of antimicrobial therapy was calculated using the following formula:

$$\text{True dose}\;\left(\mathrm{mg}/\mathrm{kg}\right):\frac{\mathrm{mg}\;\text{antimicrobial}}{\text{Body weight}\;\left(\mathrm{kg}\right)}$$

The breed/sex/age mean weight of Australian dogs was then used to calculate a proxy dose using the following formula:

$$\text{Subgroup mean imputation VetCompass Australia}\;\left(\mathrm{mg}/\mathrm{kg}\right):\frac{\mathrm{mg}\;\text{antimicrobial}}{\text{Mean weight}\;\left(\mathrm{kg}\right)}$$

The expected breed weight, published by the AKC was then used to calculate a proxy dose using the following formula:

$$\text{Imputation}‐\mathrm{AKC}\;\left(\mathrm{mg}/\mathrm{kg}\right):\frac{\mathrm{mg}\;\text{antimicrobial}}{\mathrm{AKC}\;\text{weight}\;\left(\mathrm{kg}\right)}$$

Dosing regimens were classified as ‘appropriate’ if they were within the dosing range recommended in the Australian Veterinary Prescribing Guidelines from the University of Melbourne with a buffer of ±10% (Table 2). All other doses were classified as ‘inappropriate’. The true positive and true negative rates were then calculated using the following formula:

$$\text{True positive rate}:\frac{\text{Appropriate using subgroup mean imputation}-\mathrm{VCA}\;\text{or imputation}\;\mathrm{AKC}}{\text{Actual appropriate}.\;}$$

$$\text{True negative rate}:\frac{\text{Inappropriate using subgroup mean imputation}-\mathrm{VCA}\;\text{or imputation AKC}}{\text{Actual inappropriate}}$$

**Table 2:** Minimum and maximum dose recommendations published by the University of Melbourne.^12^

	Dose (mg/kg)			
Antibiotic	Min.	Max.	Min. − 10%	Max. + 10%
Amoxycillin	11	22	9.9	24.2
Amoxycillin clavulanate	12.5	25	11.25	27.5
Cephalexin	22	30	19.8	33
Clindamycin hydrochloride	11	11	9.9	12.1
Doxycycline	5	10	4.5	11
Enrofloxacin	5	20	4.5	22
Marbofloxacin	2.75	5.5	2.475	6.05
Metronidazole	10	15	9	16.5
Pradofloxacin	3	5	2.7	5.5
Trimethoprim sulfadiazine	15	30	13.5	33

A buffer of 10% was allowed for assigning appropriateness to doses.

Statistical analyses were performed using functions in Stata 17.0.

## Results

Records for 72,089 dogs were extracted from VetCompass Australia for cohort 1. There were 250 dogs where age was not recorded. Age was less than 1 year in 10,097 records. Weight was greater than three standard deviations from the mean in 1876 records. The dataset contained 25,245 duplicate clinical records resulting in 34,621 records remaining for analysis (Table 3).

**Table 3:** Breeds and number of dogs included in cohort 1

Breed	Number of dogs
Labrador retriever	4194 (12.1%)
Jack Russell terrier	4095 (11.8%)
Staffordshire bull terrier	4069 (11.8%)
Border collie	3730 (10.8%)
Cavalier king Charles spaniel	3426 (9.9%)
German shepherd	3390 (9.8%)
Golden retriever	3312 (9.6%)
Australian cattle dog	3251 (9.4%)
Australian kelpie	2609 (7.5%)
Rottweiler	2545 (7.4%)
Total	34,621

For all breeds weight was normally distributed. The mean weight for male dogs was higher than female dogs in all breeds. Neutered dogs were generally heavier than unneutered dogs in both males and females. The weight was different for Australian Kelpies, Border Collies, Cavalier King Charles Spaniels, German Shepherd Dogs, Labrador Retrievers and Rottweilers and male Jack Russell Terriers. The only exception was female Labrador Retrievers where unneutered dogs were heavier (Tables 4 and S1). The age at which weight plateaued varied by breed, sex and neutering status (Table 4). Mean weights for animals in VetCompass were significantly higher than AKC for all breeds except Rottweilers (P < 0.001).

**Table 4:** Mean weight from VetCompass Australia database with 95% confidence interval for population weight for breed, sex, neutered status and age for the 10 most common dog breeds in Australia

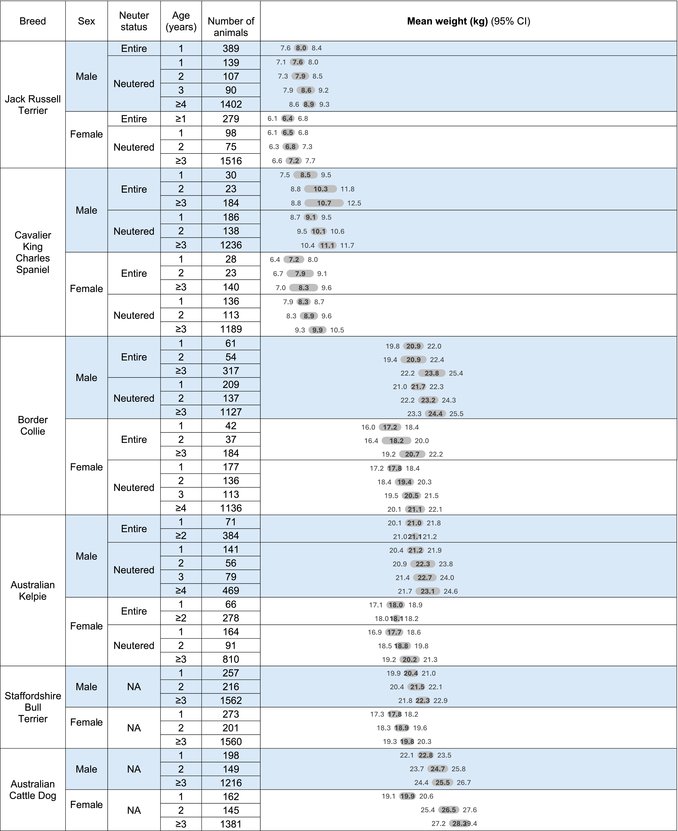
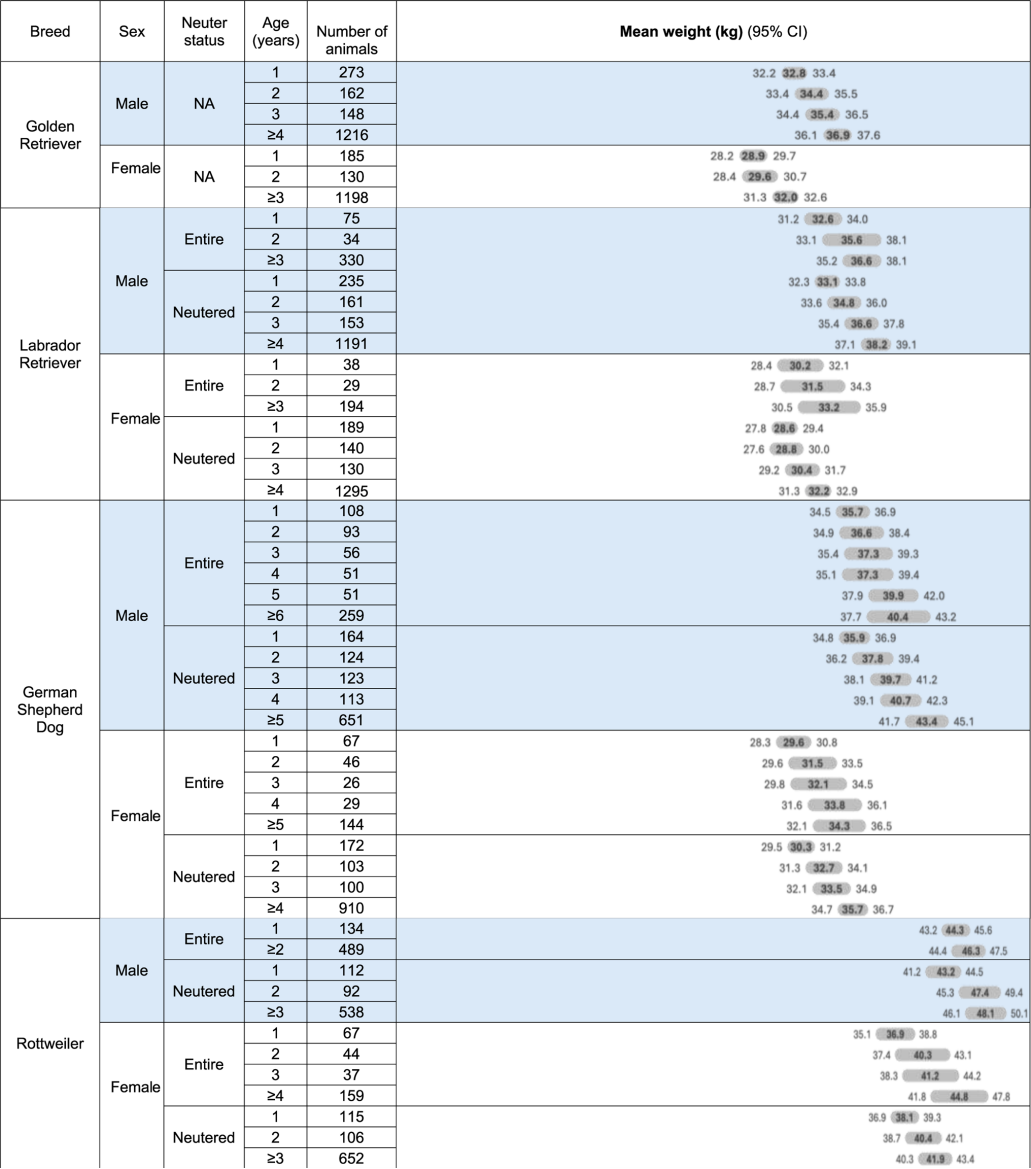

NA, not applicable as no significant difference between neutered and entire.

Subgroup mean weights were compared to multiple imputation weights and actual weights in regression models (Tables S2–S4). Subgroup mean imputation outperformed multiple imputation for breed weights after adjusting for age, sex and neutered status.

In cohort 2, 41,839 records were extracted from VetCompass Australia for dogs with age and weight recorded and of the relevant breeds. Records for 938 dogs were excluded, as body weight was greater than three standard deviations from the mean. Records for 1422 dogs were excluded as the antimicrobial used was not present in the Australian Veterinary Prescribing Guidelines or was administered intravenously, subcutaneously or intramuscularly. Records for 39,252 dogs remained for analysis of which 25,982 (66%) contained an appropriate antimicrobial dose when calculated using the actual body weight. True positive rate was 82% and true negative rate was 78% (Table 5) using VetCompass Australia weight proxies. Using AKC weight estimates, the true positive rate was 79% and the true negative rate was 61% (Table 6).

**Table 5:** Appropriateness of antimicrobial use in records when using VetCompass Australia subgroup mean body weights for age, sex and neutering status (cohort 1) for imputation and appropriateness using recorded body weight

		Subgroup mean weight (VetCompass)	
		Appropriate	Inappropriate
Actual weight	Appropriate	21,242 (82%)	4662 (18%)
	Inappropriate	3007 (22%)	10,341 (78%)

**Table 6:** Appropriateness of antimicrobial use in records when using American Kennel Club published body weights for imputation and appropriateness using recorded body weight

		Assigned weight (AKC)	
		Appropriate	Inappropriate
Actual weight	Appropriate	16,712 (79%)	4380 (21%)
	Inappropriate	4118 (39%)	6392 (61%)

Records exclude Jack Russell Terriers (weights not published by American Kennel Club).

## Discussion

Evaluating the appropriateness of veterinary drug prescriptions is reliant on patient bodyweights, an element missing in 40% of clinical records in the VetCompass Australia database.^9^ In this study, we found that the subgroup mean weights and AKC indicative breed weights had similar true positive rates (82% and 79%, respectively) but VetCompass Australia data performed better in detecting inappropriate doses (true negative rate 78% for VetCompass Australia vs 61% for AKC). The disparity between the AKC weights and the actual weights of Australian dogs likely contributed to the better overall performance. It is evident that Australian dogs are heavier than the indicative breed weights from AKC for all breeds in this study except Rottweilers (Tables 1 and 4). The AKC could be citing the expected weight of healthy dogs. Obesity is common in the Australian dog population^13^ and appears to be a growing problem globally.^14^, ^15^ In addition, weight was impacted by age and neutering status for many breeds in this study; factors that were not considered by AKC. Jack Russell Terriers, Staffordshire Bull Terriers and Golden Retrievers mean weights in this study were similar to those reported from the United Kingdom from 2019 but Rottweilers and Labradors were heavier and male German Shepherds were lighter.^16^

Missing data in electronic clinical records is a common issue for veterinary researchers.^9^, ^16^, ^17^, ^18^ Weight is consistently recorded by veterinary staff,^19^ but the method of recording this data in practice management software can either be linked to a clinical record or linked to a patient profile. When linked to a patient profile, this information is not linked to a consultation date, overrides previous weight data and is not available in the VetCompass Australia database. The missing data is therefore completely at random – that is, it is not linked to the weight of the animal or the likelihood of appropriate antimicrobial use. Multiple imputation has prevailed as the most rigorous approach to overcoming this problem. This, combined with the random nature of the missing data, allows for subgroup mean imputation. Big data sources also provide an opportunity to impute missing data to allow for population‐level studies for surveillance. This study demonstrates that we can estimate mean weights with small confidence intervals, even at the subpopulation level. Future research should investigate how recorded weight can be incorporated into VetCompass Australia as bodyweight is a powerful piece of clinical information used across veterinary research into disease monitoring and nutrition. Understanding clinical workflows is important to obtaining quality data from electronic medical records.

Assessment of appropriateness of antimicrobial dosing had similar accuracy to other data science tools created to interrogate antimicrobial prescribing in big data.^9^, ^20^ Only 66% of records in this study contained appropriate antimicrobial doses. Inappropriate dosing of antimicrobials is common in Australia.^21^ While the purpose of this study was not to investigate the appropriateness of antimicrobial dosing, further research to develop interventions to improve dosing of antimicrobial drugs is needed. This research may assist development of surveillance platforms that deliver feedback to individual practices and/or that highlight specific drugs that are inappropriately dosed.

This research has several limitations. We relied on breed as it is recorded by veterinary practices. Breed is usually designated by the owner and the veterinary team and may not always represent a purebred dog. In addition, we took steps to remove records that may have skewed our data by removing dogs with weights that seemed implausible (more than 3 standard deviations from mean), but it is possible that we removed some animals where weight was accurately recorded. Inaccuracies in breed and overcleaning of the data, however we used large numbers of animals (Table 3) to mitigate these, and any impact is likely to be small. We used a 10% buffer on antimicrobial dosing, as precise dosing in clinical practice is impacted by agent formulation and dose size, however this may have biased the results towards allowing under‐dosing and over‐dosing.

Purebreds make up a large proportion (44%) of the companion dog population in Australia^22^ and this research provides a viable means of imputing missing weight data, however expanding this work to designer breeds and mixed breeds is unlikely to be as accurate as weights are much more variable and breed standards are lacking. Cats also receive antimicrobials and one‐third of Australian households own a cat.^22^ It is important for AMS programs to encompass feline drug prescriptions and weight is also frequently missing from clinical records.^9^ Cat weight should be investigated using VetCompass Australia data.

In conclusion, average weights of Australian dogs were greater than those reported by the AKC, and age and neutering status significantly influenced average weight in many breeds. VetCompass Australia provided a useful source of animal weights, allowing calculation of average breed weights of Australian dogs that provide a simple means of imputing missing data with accuracy similar to multiple imputation for population‐level studies. Assessment of appropriateness with imputed data provides a means of overcoming missing data in VetCompass Australia.

## Conflicts of interest and sources of funding

The authors declare no conflicts of interest or sources of funding for the work presented here.

## Supporting information


**Table S1.** Multivariate generalised linear regression model of actual weight adjusted for sex and neutering status.
**Table S2.** Linear regression model of actual weight adjusted for age, neutering status, sex and breed.
**Table S3.** Linear regression model of weight using multiple imputation adjusted for age, neutering status, sex and breed.
**Table S4.** Linear regression model of weight using subgroup mean imputation adjusted for age, neutering status, sex and breed.

## Data Availability

The data that support the findings of this study are available on request from the corresponding author. The data are not publicly available due to privacy or ethical restrictions.
