# Supplementary material for: Use of VetCompass Australia data to estimate body weight for antimicrobial stewardship in Australian dogs
Source: Aust Vet J. 2026 Jun 4;104(10):674–80. doi: 10.1111/avj.70099 (PMC13631542; doi:10.1111/avj.70099)
Supplement: Supplementary file 1 [file AVJ-104-674-s001.docx]

Supplementary table 1. Multivariate generalised linear regression model of actual weight adjusted for sex and neutering status

|  |  | Coefficient | 95% conf. interval | P |
| --- | --- | --- | --- | --- |
| Australian Cattle Dog | Neutered status  Entire  Neutered | Ref  0.31 | -0.1, 0.73 | 0.139 |
|  | Sex  Female  Male | Ref  2.8 | 2.5,3.2 | <0.001 |
| Australian Kelpie | Neutered status  Entire  Neutered | Ref  1.4 | 1.1, 1.8 | <0.001 |
|  | Sex  Female  Male | Ref  2.8 | 2.4,3.1 | <0.001 |
| Border Collie | Neutered status  Entire  Neutered | Ref  0.92 | 0.51, 1.3 | <0.001 |
|  | Sex  Female  Male | Ref  3.3 | 2.97,3.6 | <0.001 |
| Cavalier King Charles Spaniel | Neutered status  Entire  Neutered | Ref  0.79 | 0.50, 1.1 | <0.001 |
|  | Sex  Female  Male | Ref  1.6 | 1.4, 1.8 | <0.001 |
| German Shepherd | Neutered status  Entire  Neutered | Ref  1.2 | 0.67, 1.7 | <0.001 |
|  | Sex  Female  Male | Ref  5.7 | 5.3, 6.2 | <0.001 |
| Labrador Retriever | Neutered status  Entire  Neutered | Ref  -0.56 | -1.0, -0.06 | 0.028 |
|  | Sex  Female  Male | Ref  4.6 | 4.2, 5.0 | <0.001 |
| Rottweiler | Neutered status  Entire  Neutered | Ref  0.83 | 0.21, 1.5 | 0.009 |
|  | Sex  Female  Male | Ref  6.0 | 5.4, 6.6 | <0.001 |
| Golden Retriever | Neutered status  Entire  Neutered | Ref  0.20 | -0.29, 0.70 | 0.421 |
|  | Sex  Female  Male | Ref  3.9 | 3.5, 4.3 | <0.001 |
| Staffordshire Bull Terrier | Neutered status  Entire  Neutered | Ref  -0.21 | -0.53, 0.10 | 0.184 |
|  | Sex  Female  Male | Ref  2.7 | 2.4, 3.0 | <0.001 |
| Jack Russell Terrier | Neutered status  Entire  Neutered | Ref  0.45 | 0.27, 0.63 | <0.001 |
|  | Sex  Female  Male | Ref  1.2 | 1.1, 1.3 | <0.001 |

Supplementary table 2. Linear regression model of actual weight adjusted for age, neutering status, sex and breed.

|  | Coefficient | 95% conf. interval | P |
| --- | --- | --- | --- |
| Age | 0.33 | 0.35, 0.34 | <0.001 |
| Neutered status  Entire  Neutered | Ref  0.68 | 0.55, 0.81 | <0.001 |
| Sex  Female  Male | Ref  3.5 | 3.4, 3.6 | <0.001 |
| Breed  Australian Cattle Dog  Australian Kelpie  Border Collie  Cavalier King Charles Spaniel  German Shepherd Dog  Golden Retriever  Jack Russell Terrier  Labrador Retriever  Rottweiler  Staffordshire Bull Terrier | Ref  -2.4  -1.5  -13.2  12.5  9.1  -16.0  9.9  19.9  -3.5 | -2.6, -2.2  -1.7, -1.2  -13.4, -12.9  12.3, 12.8  8.8, 9.3  -16.3, -15.8  9.6, 10.1  19.7, 20.1  -3.7, -3.2 | <0.001  <0.001  <0.001  <0.001  <0.001  <0.001  <0.001  <0.001  <0.001 |

Supplementary table 3. Linear regression model of weight using multiple imputation adjusted for age, neutering status, sex and breed.

|  | Coefficient | 95% conf. interval | P |
| --- | --- | --- | --- |
| Age | 0.18 | 0.14, 0.21 | <0.001 |
| Neutered status  Entire  Neutered | Ref  0.39 | 0.02, 0.76 | 0.041 |
| Sex  Female  Male | Ref  1.83 | 1.5, 2.1 | <0.001 |
| Breed  Australian Cattle Dog  Australian Kelpie  Border Collie  Cavalier King Charles Spaniel  German Shepherd Dog  Golden Retriever  Jack Russell Terrier  Labrador Retriever  Rottweiler  Staffordshire Bull Terrier | Ref  -1.1  -0.83  -6.7  6.2  4.5  -8.2  5.0  10.2  -1.7 | -1.7, -0.54  -1.5, -0.19  -7.3, -6.1  5.6, 6.9  3.8, 5.1  -8.9, -7.5  4.5, 5.6  9.6, 10.9  -2.4, -1.0 | <0.001  <0.001  <0.001  <0.001  <0.001  <0.001  <0.001  <0.001  <0.001 |

Supplementary table 4. Linear regression model of weight using subgroup mean imputation adjusted for age, neutering status, sex and breed.

|  | Coefficient | 95% conf. interval | P |
| --- | --- | --- | --- |
| Age | 0.18 | 0.18, 0.19 | <0.001 |
| Neutered status  Entire  Neutered | Ref  0.46 | 0.42, 0.50 | <0.001 |
| Sex  Female  Male | Ref  3.2 | 3.2, 3.2 | <0.001 |
| Breed  Australian Cattle Dog  Australian Kelpie  Border Collie  Cavalier King Charles Spaniel  German Shepherd Dog  Golden Retriever  Jack Russell Terrier  Labrador Retriever  Rottweiler  Staffordshire Bull Terrier | Ref  -1.8  -0.93  -12.6  14.2  10.0  -15.0  10.6  21.2  -2.2 | -1.9, -1.7  -1.0, -0.86  -12.6, -12.5  14.1, 14.3  10.0, 10.1  -15.1, -15.0  10.5, 10.6  21.1, 21.2  -2.2, -2.1 | <0.001  <0.001  <0.001  <0.001  <0.001  <0.001  <0.001  <0.001  <0.001 |
